# Density estimation of tiger and leopard using spatially explicit capture–recapture framework

**DOI:** 10.7717/peerj.10634

**Published:** 2021-02-17

**Authors:** Tahir Ali Rather, Sharad Kumar, Jamal Ahmad Khan

**Affiliations:** 1 Department of Wildlife Sciences, Aligarh Muslim University, Aligarh, Uttar Pradesh, India; 2 The Corbett Foundation, Mumbai, Maharashtra, India

**Keywords:** Tiger, Leopard, Population density, Camera trapping, SECR, Bandhavgarh

## Abstract

The conservation of large carnivores often requires precise and accurate estimates of their populations. Being cryptic and occurring at low population densities, obtaining an unbiased population estimate is difficult in large carnivores. To overcome the uncertainties in the conventional capture–recapture (CR) methods used to estimate large carnivore densities, more robust methods such as spatially explicit capture-recapture (SECR) framework are now widely used. We modeled the CR data of tiger (*Panthera tigris tigris*) and leopard (*Panthera pardus fusca*) in the SECR framework with biotic and abiotic covariates likely believed to influence their densities. An effort of 2,211 trap nights resulted in the capture of 33 and 38 individual tigers and leopards. A total of 95 and 74 detections of tigers and leopards were achieved using 35 pairs of camera traps. Tiger and leopard density were estimated at 4.71 ± 1.20 (3.05–5.11) and 3.03 ± 0.78 (1.85–4.99) per 100 km^2^. Our results show that leopard density increased with high road density, high terrain ruggedness and habitats with high percentage of cropland and natural vegetation. The tiger density was positively influenced by the mosaic of cropland and natural vegetation. This study provides the first robust density estimates of tiger and leopard within the study area. Our results support the notion that large carnivores can attain moderate densities within human-dominated regions around protected areas relying on domestic livestock. Broader management strategies aimed at maintaining wild prey in the human-dominated areas around protected areas are necessary for large and endangered carnivores’ sustenance in the buffer zones around protected areas.

## Introduction

Large carnivores are cryptic, highly mobile, and often occur at low densities, and thus, it is difficult to correctly estimate their population estimates (Garshelis, 1992; Karanth, 1995; Boulanger et al., 2004). In recent years, the improvements in the use of capture–recapture (CR) methods employing remotely operated camera traps (Karanth, 1995) or from DNA samples (Woods et al., 1999) have enabled researchers to estimate the population parameters of cryptic carnivores with high certainty. However, conventional CR methods provide estimates of population size (N) and not population density (D) (Obbard, Howe & Kyle, 2010). The estimates of animal abundances make biological sense only when the sampled area (A) is precisely known, and all animals have spatially homogenous detection probability (Parmenter et al., 2003). Thus by dividing the abundances by total A, one can derive estimates of the D. In geographically open populations, the estimates of animal abundances (N) are overestimated because only a portion of the animal home ranges is in the array of camera traps. Thus, a small number of animals are available for sampling (White et al., 1982). Dice (1938) termed this effect of positive bias as the edge effect. It remains one of the major problems regarding the correct estimation of carnivore densities (Karanth et al., 2006; Kendall et al., 2008). Most often, to correct for positive bias, a buffer strip of width (W) based on Mean Maximum Distance Moved (MMDM) by recaptured animals or half of the MMDM is added around the array of the camera traps to estimate effective trap area (Karanth & Nichols, 2002). Field studies have shown that such inter-trap distances are underestimates of the actual distances moved by re-captured animals because they are truncated at trap locations (Efford, 2004; Obbard, Howe & Kyle, 2010). The accurate strip widths can be obtained only if radio-telemetry data are available for recaptured animals.

To overcome the uncertainties in conventional CR methods caused by the edge effect and spatially heterogeneous detection probabilities of animals due to the movement, Efford (2004) introduced a method that directly estimates animal densities from CR data without the assumption of geographical closure or estimating the effective trap area. In his approach, Efford (2004) combined CR and conventional distance sampling (Burnham, Anderson & Laake, 1980) in what is called Spatially Explicit Capture–Recapture (SECR) methods. SECR method estimates three model parameters: g_0_ the fitted detection probability function, sigma (σ) the spatial extent over which capture probability declines, and D, which is defined as the intensity of spatial point process describing the locations of home range centers (Efford, 2004). Since its inception in 2004, more flexible and maximum likelihood-based estimators of density have been developed (Borchers & Efford, 2008). Bayesian-based SECR modeling approaches have also been developed in parallel with the maximum likelihood-based SECR approaches (Royle & Young, 2008; Gardner, Royle & Wegan, 2009; Royle et al., 2009).

In India, the estimation of abundances of tigers using camera traps started as early as the 1990s, first in Nagarahole Tiger Reserve (Karanth, 1995) under a closed CR framework (Otis et al., 1978; White et al., 1982). The modeling approach adopted by Karanth (1995) soon led to the identification of the issues related to the trap-spacing, geographical, and statistical population closure assumptions, model selection, and density estimations. These issues were adequately addressed in the subsequent refinements introduced periodically (Karanth & Nichols, 1998, 2002). Since then, most of the studies employing camera traps to estimate carnivore abundances (Karanth et al., 2004a, 2004b; 2006; Kawanishi & Sunquist, 2004; Simcharoen et al., 2007) were based on the approaches developed by Karanth & Nichols (1998, 2000) and their modifications (O’Brien, Kinnaird & Wibisono, 2003; Wegge, Pokheral & Jnawali, 2004; Johnson et al., 2006). Camera traps have been widely used to estimate population abundances of large carnivores that could be identified based on natural markings such as tigers (Karanth, 1995; Karanth & Nichols, 1998; Karanth et al., 2006), ocelots (Trolle & Kéry, 2003, 2005), jaguars (Silver et al., 2004; Maffei, Cuéllar & Noss, 2004) and leopards (Balme, Hunter & Slotow, 2009; Harihar, Pandav & Goyal, 2009; Karanth et al., 2006; Athreya et al., 2013).

A variety of ecological factors influences predator densities. Karanth et al. (2004b) reported a functional relationship between large predators’ abundances and their prey under a wide range of ecological conditions. Prey density is one of the important factors determining the abundance of tigers (Karanth et al., 2004b) and leopards (Carbone, Pettorelli & Stephens, 2011). Similar studies have found multiple correlates associated with tiger and leopard densities. Factors such as forest type, vegetation cover and reserve size (Sunarto et al., 2015; Havmøller et al., 2019), habitat connectivity (Joshi et al., 2013), livestock depredation, and human settlements (Harihar & Pandav, 2012; Karanth et al., 2011), are regarded as most influential covariates influencing the large carnivore densities.

Here, we used the CR data to estimate the densities of tiger and leopard with the habitat covariates likely believed to influence tiger and leopard occurrence in human-dominated regions. We modeled the data in a SECR framework to test the hypothesis on the ecological and anthropogenic drivers of tiger and leopard densities in the human-dominated buffer zone of the Bandhavgarh Tiger Reserve in Central India. More specifically, we investigated the influence of different habitat types on the density estimation of tiger and leopard.

## Materials and Methods

### Study area

**Figure 1:**
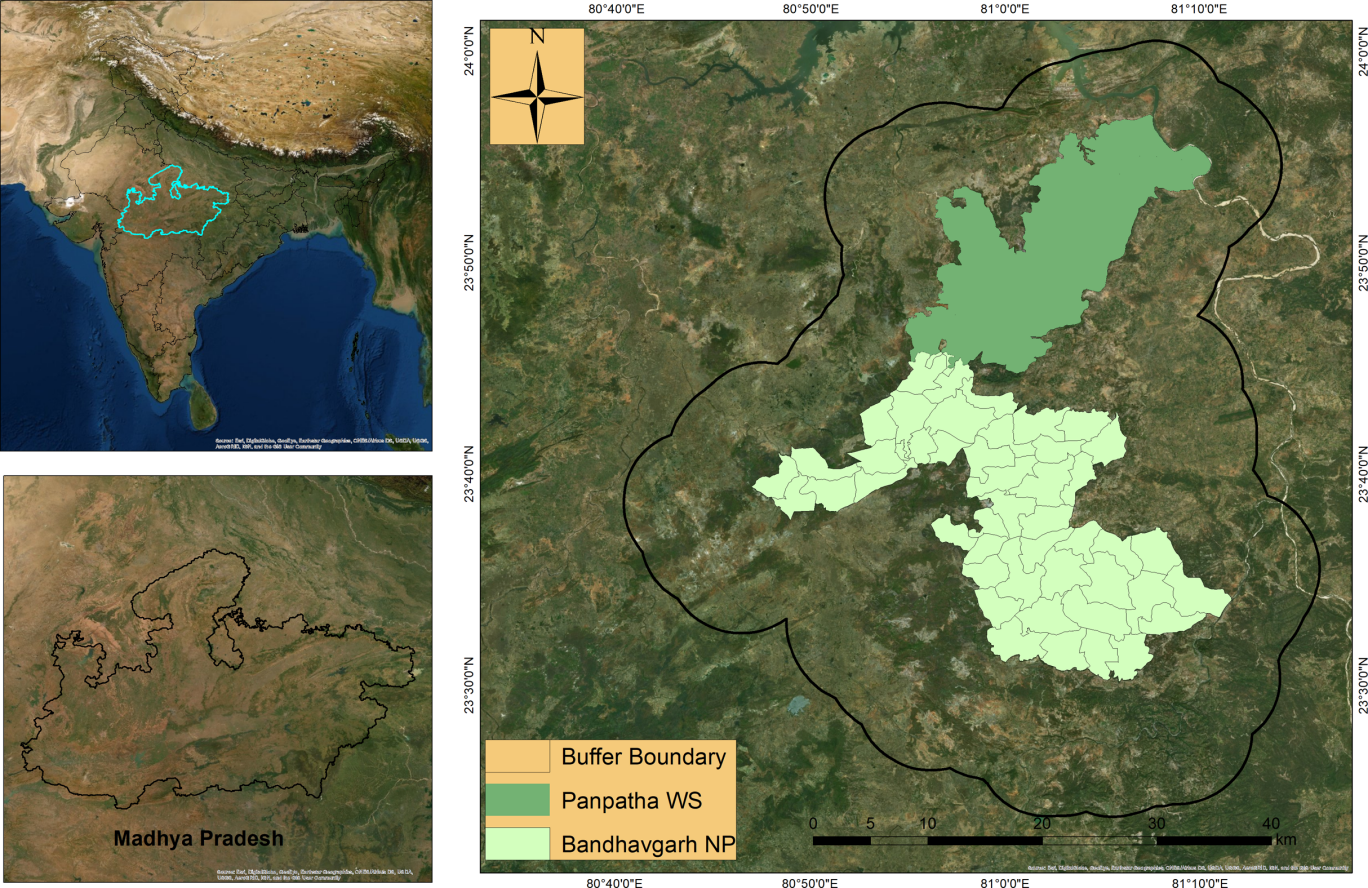
Location of Bandhavgarh Tiger Reserve, Madhya Pradesh, India. Location of Bandhavgarh Tiger Reserve, Madhya Pradesh, India. The Panpatha Wildlife Sanctuary (PWS) in the north and Bandhavgarh National Park (BNP) in the south constitute the core of the Bandhavgarh Tiger Reserve. ESRI DigitalGlobe, GeoEye, Earthstar Geographics, CNESAirbus DS, USGS, AeroGRID, IGN and GIS User Community.

Bandhavgarh Tiger Reserve (BTR) is located between 23° 27′ 00″ to 23° 59′ 50″North latitude and 80° 47′ 75″ to 81° 15′ 45″ East longitude in Central India. BTR consists of two Protected areas: Bandhavgarh National Park (BNP) in the south and the Panpatha Wildlife Sanctuary (PWS) in the north (Fig. 1). BNP and PWS constitute the core zone of the reserve, having a combined area of 716 km^2^. The surrounding buffer zone has an area of 820 km^2^, adding the total area of the reserve to 1,536 km^2^. A more detailed account of the study area is available in Rather, Kumar & Khan (2020). The vegetation comprises moist peninsular low-level sal forest, northern dry mixed deciduous forest, dry deciduous scrub, dry grassland, and west Gangetic moist mixed deciduous forest (Champion & Seth, 1968). Sal and sal mixed forests occur in the major portion of the reserve. Besides tiger and leopard, sloth bear (*Melursus ursinus*), Indian wolf (*Canis lupus*), Asiatic wild dogs (*Cuon alpinus*), and striped hyaena (*Hyaena hyaena*) are notable carnivore species occurring within the reserve. Major prey species include chital (*Axis axis*), sambar (*Rusa unicolor*), Indian guar (*Bos gaurus*), barking deer (*Muntiacus munjtak*), Indian gazelle (*Gazella bennetti*), four-horned antelope (*Tetracerus quadricornis*), and Indian blue bull (*Boselaphus tragocamelus*). Most of the reserve has a flat to the gentle slope with an average elevation of 570 m (asl). The annual average rainfall in reserve is reported to be 1,173 mm, most of which occur in the monsoon season. The reserve has a fair road network presence, allowing easy and adequate camera traps placement along roads and trails.

### Camera trap survey

We conducted extensive reconnaissance within the study area to identify the most optimal locations for setting up camera traps to maximize the probability of detecting as many individuals and obtain as many photo-captures of the individual tigers and leopards as possible. We selected trap location for camera traps based on secondary evidence such as pugmarks, scats, scratches, marking on trees, etc. The average inter-trap distance was 856 meters to avoid leaving any large gaps between the successive camera traps (Supplemental Information S1). At any time, 20–25 pairs of camera traps Cuddeback^™^ model C1 (total 35 pairs) remained active within the study area yielding 2,211 trap nights at 220 sites. Camera traps were placed along roads and trails at a distance of 5–10 m from the center of roads or trails. At each location, a pair of camera trap was tied to the trees on either side at the height of 30–40 cm from the ground. The delay between the successive photo-captures was set to 5 seconds to increase the chances of photo-capturing animals traveling either in groups or mothers accompanying cubs. Camera traps were kept active 24 h at the stations where the theft risk was minimal and checked daily. No baits were used to lure the animals towards camera traps. The study area was divided into three blocks corresponding to the three buffer ranges of the reserve. Camera traps were placed within each sampling block for eight consecutive days before being moved to new sites. Camera traps were set for two months in each sampling blocks between 2016 and 2017.

### Habitat covariates

We derived a set of eight habitat covariates to model the density of the tiger and leopard (Supplemental Information S2). The habitat variables were based on a recent study predicting tiger and leopard occurrence in the study area (Rather, Kumar & Khan, 2020). We used land use land cover map of the study area prepared by the Indian Institute of Remote Sensing (IIRS; www.iirs.gov.in) and derived eight habitat variables at the spatial resolution of 1km. We used a moving window analysis in ArcGIS (version 10.3) and extracted the habitat variables for each camera trap location. We used a circular buffer of 1,000 m radius around each camera trap location and calculated the percentage of available habitat types. We used road and river density calculated within the radius of 1,000 m instead of distance to roads and rivers due to the high concentration of roads within the study area. The density of roads, percentage of human settlements, and percentage of degraded forests (mosaic of croplands and natural vegetation) within the spatial extent of 1,000 m were considered as a proxy to human disturbance.

### Individual identification of tigers and leopards

Each photograph was carefully examined for the shape and pattern of natural markings; stripes in tigers and rosettes in leopards using both right and left flanks, limbs, tails, and forequarters (Schaller, 1967; McDougal, 1977; Karanth, 1995; Franklin et al., 1999). Tigers and leopards were individually identified and given a unique identity. Subsequent identification of new individuals was achieved by comparing and matching each photograph with the known and uniquely identified tigers and leopards. Respective capture histories of identified tigers and leopards and their trap locations were constructed in the form of two separate text delimited files (Supplemental Informations S3, S4 and S5). The analysis was done using the package ‘secr’ version 4.3.1 (Efford, 2020) implemented in R (R Core Team, 2018).

### Buffer width and detection functions

Since there was a reasonably suitable habitat around camera traps, so it was reasonable to pay attention to the area immediately around the camera traps by specifying a habitat buffer. In SECR, the buffer width is not critical as long as it is wide enough so that the animals occupying edges have virtually zero chances of appearing in the sample. The buffer widths were based on the average inter-trap distances (initial sigma) moved by animals. In SECR, the buffer widths are multiplies of initial sigma, and the fit is assessed when the density estimates do not change with an increase in buffer widths. In this analysis, the average inter-trap distance (initial sigma) moved by tigers and leopards was 1,581 and 1,574 m. The buffer widths were calculated by multiplying the initial sigma values for each detection function until no change in density was observed.

### Density models

We fitted both the null models (D ~ 1, lambda0 ~ 1, sigma ~ 1) and the models with habitat covariates (D ~ cov, lambda0 ~ 1, sigma ~ 1) to model the distribution of animal activity centres within the defined state-space S. Among null models we tested three models each for tiger and leopard as: (D ~ 1, lambda0 ~ 1, sigma ~ 1), (D ~ 1, lambda0 ~ b, sigma ~ 1), (D ~ 1, lambda0 ~ bk, sigma ~ 1). We tested different hypotheses by studying the effects of habitat covariates on the tiger and leopard density. First, we defined a null model (D ~ 1, lambda0 ~ 1, sigma ~ 1). Subsequently, we assessed the effect of the following covariates on the density of tiger and leopard: (i) sal dominated forests with thick vegetation cover, (ii) moist deciduous forests, (iii) dry deciduous forests, (iv) degraded forests, (v) road density, (vi) river density, (vii) human settlements and (viii) terrain ruggedness. We expected tiger and leopard density to increase with sal-dominated forests, moist deciduous forests, and dry deciduous forests as these forest types are associated with high prey presence. We also expected density to be positively correlated with rugged terrain due to the low anthropogenic influence in rugged topographical areas. We also expected tiger and leopard densities to be negatively correlated with disturbed habitats (degraded forests). We set a maximum buffer of 8 km based on initial sigma around each trap site based on rigged density estimates. We calculated the Akaike Information Criterion (AIC) for each competing model and selected the best model based on the difference between AIC scores (ΔAIC).

## Results

### Population estimates

We obtained 95 detections of 33 individual tigers and 74 detections of 38 individual leopards over 2,211 trap nights in three sampling blocks. Tigers and leopards were detected in 87 and 70 camera trap stations, respectively. The overall capture probability (*M* (*t*+1)/*n*) of leopards (0.56) was slightly higher than tigers (0.45). The cumulative number of detected individuals (tigers and leopards) on each occasion (*t*) was pooled together for all sampling blocks to estimate sampling adequacy (Supplemental Information S6). The camera trap arrangement for eight consecutive days at each site before moving to new sites used in this study was adequate for sampling tigers. The unique individuals captured did not reach asymptote as promptly as tigers in the case of leopards (Supplemental Information S6). Sharma et al. (2010) found that camera traps failed to detect all known tigers in Kanha Tiger Reserve, even after prolonged sampling. Thus it is challenging to achieve total counts in natural populations using camera traps even after intensive sampling efforts (Sharma et al., 2010). We achieved a sampling coverage of over 500 km^2^ in the reserve buffer zone (836 km^2^). Thus the trapping area met the size requirement as recommended by Silver et al. (2004), Maffei & Noss (2008) and Sharma et al. (2010) for estimating the densities of tigers and leopards.

About 40% of trap-revealed movements in tigers were within the range of 1,000–2,000 m, and 25% of trap-revealed movements in leopards were within the range of 3,000 m; thus, there is a high probability that peripheral individuals had a good chance of being photo-captured even if their home ranges were centered outside the plotted area.

### Fitting SECR models and density estimations of tiger and leopard

The models were fitted using the buffer W of 7,908 and 7,870 meters for HN, EX and HR detection functions under null models to estimate the densities of tigers and leopards, respectively. We estimated densities using three model specifications: a null and permanent global learned response model (b) and a permanent detector-specific learned response (bk) model. The Null model assumes that detection of all individuals at all camera traps is governed by the same detection versus distance curve and is represented as:

(g0 ~1, sigma ~ 1)

The learned response model or behavioral response model (b) assumes that detectors (camera traps) incite a behavioral response in individuals after their first encounter with detectors. Thus the probability of capturing an individual on any later occasion is affected (Otis et al., 1978). Moreover, the learned response may be specific to the detector location (bk) rather than generally applying across all detectors. We considered the possibility of either sort of induced behavioral response in tigers and leopards.

The density of the tiger and leopard under the null model was estimated to be 3.28 ± 0.65 (Mean ± SE) per 100 km^2^ and 4.04 ± 0.83 (Mean ± SE) (Table 1). Formal model comparisons based on AIC placed the detection functions with longer tails (EX) ahead of (HN) in tiger (Table 2). However, the density estimates from the HN detection function reached a plateau fairly promptly with increasing buffer W (Supplemental Information S7) compared to negative exponential (EX) and hazard rate (HR). The AIC differences between the models using different detection functions (null models) in the tiger were also very small (Table 2). The density estimates from the null model (D~1 g0~1 sigma~1) and behavioral response models (D~1 g0~b sigma~1; D~1 g0~bk sigma~1) were not noticeably larger than each other (Table 1). The null models (D~1 g0~b sigma~1) in the tiger and leopard had the lowest AIC value in comparison to the behavioral response models (b and bk) (Table 3). The density estimations are slightly higher in leopards from the EX detection function under the null model (Table 4). The formal AIC comparison indicated the HN detection curve model as the best model (Table 2). The density of the leopard was estimated slightly lower under the behavioral response model (b) compared to the null model and (bk) model (Table 1).

**Table 1:** Comparison of density estimates in tiger and leopard using null, permanent global learned response model (b), and permanent detector-specific learned response (bk) models.

Species	Model	Parameters	Estimate	SE.estimate	lcl	ucl
Tiger	Null	D	3.28	0.65	2.2	4.81
		g0	0.174	0.03	0.11	0.26
		sigma	1,197.46	129.62	969.15	1,479.56
	B	D	3.73	0.90	2.34	5.95
		g0	0.26	0.10	0.11	0.50
		sigma	935.44	180.59	642.97	1,360.94
	bk	D	3.15	0.66	2.08	4.81
		g0	0.025	0.025	0.04	0.15
		sigma	1,824.91	244.35	1,405.32	2,369.78
Leopard	null	D	4.04	0.83	2.72	6.01
		g0	0.039	0.008	0.02	0.06
		sigma	2,136.74	236.45	1,721.26	2,652.52
	b	D	3.20	0.69	2.11	4.86
		g0	0.05	0.01	0.02	0.10
		sigma	2,342.30	351	1,748.00	3,138.66
	bk	D	4.18	0.91	2.07	6.38
		g0	0.039	0.07	1.87	5.06
		sigma	2,254.03	264	1,791.03	2,836.83

**Note:** The estimated parameters include the density (D), detection probability (g0) and sigma, the spatial extent over which capture probability declines.

**Table 2:** Summary of AIC values for density estimates of tiger and leopard calculated using half normal (HN), negative exponential (EX) and hazard rate (HR) detection functions.

Species	Det Fn	Model	npar	logLik	AIC	AICc	dAIC	AICcwt
Tiger	HN	D~1 g0~1 sigma~1	3	−279.320	565.13	565.96	0.490	0.401
	EX	D~1 g0~1 sigma~1	3	−279.323	564.64	565.47	0.000	0.513
	HR	D~1 g0~1 sigma~1 z~1	4	−279.826	569.65	569.08	3.608	0.845
Leopard	HN	D~1 g0~1 sigma~1	3	−233.293	472.58	473.29	0.000	0.425
	EX	D~1 g0~1 sigma~1	3	−233.416	472.83	473.53	0.245	0.376
	HR	D~1 g0~1 sigma~1 z~1	4	−232.805	473.61	474.82	0.1529	0.198

**Note:** The AIC differences between the models are within small ranges and thus no model is superior to another.

**Table 3:** Summary of AIC values for density estimates of tiger and leopard calculated using null model, permanent global learned response model (b), and, permanent detector-specific learned response (bk) models.

Species	Model	npar	Det Fn	logLik	AIC	AICc	dAIC	AICcwt
Tiger	D~1 g0~1 sigma~1	3	EX	−279.32	565.47	565.47	0.00	1
	D~1 g0~bk sigma~1	4	EX	−458.47	924.95	926.38	360.90	0
	D~1 g0~b sigma~1 z~1	4	EX	−460.28	928.56	929.99	364.51	0
Leopard	D~1 g0~1 sigma~1	3	HN	−233.293	472.58	473.29	0.000	1
	D~1 g0~bk sigma~1	4	HN	−374.687	757.37	758.58	285.29	0
	D~1 g0~b sigma~1 z~1	4	HN	−376.069	760.13	761.35	288.05	0

**Note:** In models (D~1 g0~bk sigma~1) and (D~1 g0~bk sigma~1) the detection probability is conditional on learned response (b) and detector specific response (bk).

**Table 4:** Density estimation of tiger and leopard under the assumptions of null model for three detection functions.

Species	Dt Fn	Parameters	Estimate	SE	lcl	ucl
Tiger	HN	D	3.12	0.60	2.15	4.54
		g0	0.069	0.01	0.04	0.09
		sigma	1,959.56	160.95	1,668.65	2,301.20
	EX	D	3.28	0.65	2.2	4.81
		g0	0.174	0.03	0.11	0.26
		sigma	1,197.46	129.62	969.15	1,479.56
	HR	D	3.12	0.60	2.12	4.56
		g0	0.05	0.02	0.02	0.10
		sigma	2,752.76	863.95	1,509.62	5,019.57
Leopard	HN	D	4.04	0.83	2.72	6.01
		g0	0.039	0.008	0.02	0.06
		sigma	2,136.74	236.45	1,721.26	2,652.52
	EX	D	4.41	0.94	2.92	6.06
		g0	0.102	0.027	0.05	0.16
		sigma	1,268.13	177.79	964.73	1,666.94
	HR	D	4.28	0.93	2.05	6.51
		g0	0.032	0.010	0.01	0.06
		sigma	2,692.08	709.62	1,619.84	4,474.06

**Note:** HN = half normal, EX = negative exponential, and HR = hazard rate. The three model parameters calculated are D = density per 100 km^2^, g0 = probability detection function and sigma = distance over which probability of detection decreases.

The density estimates based on the models with covariates were slightly higher 4.71 ± 1.20 (3.05–5.11) per 100 km^2^ for tiger and lower for leopards 3.03 ± 0.78 (1.85–4.99) compared to null models (Tables 5 and 6). The models, including degraded forest type and moist deciduous forest type, were the most parsimonious models based on AIC weight for tiger and leopard (Tables 5 and 6).

**Table 5:** Density estimation and model selection of tiger based on AIC comparisons. The habitat variables were calculated using the land use land cover (LULC) map of the study area at the spatial resolution of 1 km. The habitat variables were calculated as the percentage of available habitat within the spatial radius of 1 km from the camera trap locations. The density estimates are calculated as the number of animals per 100 km^2^. mdec = moist deciduous forests, rd1k = road density within 1 km radius of the camera traps, river = permanent water bodies within 1 km radius of camera traps, sal = sal dominated habitat, degraded = mosaic of croplands and natural vegetation, set = permanent human settlement, drydec = dry deciduous habitat, and rug = topographic ruggedness.

Model	Detfn	LogLik	Density	SE	lcl	ucl	AIC	AICc	dAIC	AICwt
D~degraded lambda0~1 sigma~1	HNN	−272.49	4.71	1.20	3.05	5.11	552.99	554.42	0.00	0.9916
D~rd1k lambda0~1 sigma~1	HNN	−277.26	1.37	0.67	0.54	3.39	562.53	563.96	9.54	0.0084
D~rug lambda0~1 sigma~1	HNN	−278.49	2.82	0.87	1.89	4.23	564.98	566.40	11.98	0.0000
D~set lambda0~1 sigma~1	HNN	−278.66	3.00	0.86	2.02	4.45	565.33	566.76	12.34	0.0000
D~drydec lambda0~1 sigma~1	HNN	−278.92	2.72	0.36	1.73	4.28	565.85	567.28	12.86	0.0000
D~river lambda0~1 sigma~1	HNN	−279.20	2.79	0.58	1.76	4.40	566.41	567.84	13.42	0.0000
D~sal lambda0~1 sigma~1	HNN	−279.56	3.20	1.00	1.76	5.83	567.13	568.56	14.13	0.0000
D~mdec lambda0~1 sigma~1	HNN	−279.58	3.08	0.36	2.07	4.58	567.16	568.58	14.16	0.0000

**Table 6:** Density estimation and model selection of leopard based on AIC comparisons. Models were run using only single covariates on density (D) while keeping lambda0 and sigma constant. The habitat variables were calculated using the land use land cover (LULC) map of the study area at the spatial resolution of 1 km. The habitat variables were calculated as the percentage of available habitat within the spatial radius of 1 km from the camera trap locations. The density estimates are calculated as the number of animals per 100 km^2^. mdec = moist deciduous forests, rd1k = road density within 1 km radius of the camera traps, river = permanent water bodies within 1 km radius of camera traps, sal = sal dominated habitat, degraded = mosaic of croplands and natural vegetation, set = permanent human settlement, drydec = dry deciduous habitat, and rug = topographic ruggedness.

Model	Detfn	LogLik	Density	SE	lcl	ucl	AIC	AICc	dAIC	AICwt
D~mdec lambda0~1 sigma~1	HHN	−229.52	3.03	0.78	1.85	4.99	467.05	468.26	0.00	0.567
D~rd1k lambda0~1 sigma~1	HHN	−230.38	8.41	2.69	4.55	15.5	468.76	469.98	1.71	0.240
D~river lambda0~1 sigma~1	HHN	−231.70	2.93	0.83	1.70	5.07	471.40	472.61	4.35	0.064
D~sal lambda0~1 sigma~1	HHN	−232.12	2.41	0.94	1.15	5.04	472.25	473.46	5.20	0.042
D~degraded lambda0~1 sigma~1	HHN	−232.35	5.08	1.14	3.28	7.87	472.71	473.92	5.66	0.033
D~set lambda0~1 sigma~1	HHN	−232.81	3.71	0.89	2.43	5.66	473.62	474.83	6.57	0.021
D~drydec lambda0~1 sigma~1	HHN	−232.93	3.58	0.83	2.28	5.63	473.86	475.07	6.81	0.018
D~rug lambda0~1 sigma~1	HNN	−233.40	4.44	2.27	2.85	6.91	474.81	476.02	7.76	0.011

## Discussion

### Correlates of tiger and leopard density

The density of tigers and leopards were calculated using maximum likelihood-based SECR approaches under two broad model categories. The density estimates from the first category models (null and behavioral response models) in the tiger were not noticeably larger than each other. The AIC differences between the models using different detection functions (null model) in the tiger were also very small. No obvious inferences could be drawn based on null models. The leopard density was estimated slightly lower under the behavioral response model (b) than the null and (bk) model. The null model category performed all equal, and no meaningful conclusions could be achieved.

Thus, we created a second model category where the density was modeled with habitat covariates hypothesized to influence tiger and leopard densities in human-dominated habitats. We expected densities to be positively correlated with undisturbed habitats such as sal-dominated habitats and moist and dry deciduous forests. The undisturbed habitats are associated with high prey abundance (wild prey) and low anthropogenic influence. The densities of tigers and leopards have been reported to depend on prey biomass (Karanth et al., 2004b; Carbone & Gittleman, 2002; Khorozyan, Malkhasyan & Abramov, 2008; Carbone, Pettorelli & Stephens, 2011). Although tiger density was positively correlated with sal dominated habitats; however, contrary to our expectations, the tiger density was also positively associated with disturbed habitats. The positive correlation of tiger density in degraded habitats may likely be due to livestock availability in such habitats. In a recent study, the density of Asiatic lions (*Panthera leo persica*) was found to be negatively correlated with their natural prey in the tourism zone (disturbed) due to the assured food provisioning (Gogoi et al., 2020). A recent diet analysis indicates that more than 40% of the seasonal biomass consumed by tigers in the buffer zone of BTR was represented by livestock (Rather, 2020) compared to only 5% biomass consumption of livestock in the core zone of the reserve (Navaneethan et al., 2019). The density of leopards was positively influenced by road density, rugged terrain, and degraded habitats. The AIC comparison indicated the model with a moist deciduous forest to be the best density estimation model of leopard (Table 6). The high density of leopards relative to road density indicates the frequent use of forest roads as regular travel routes by leopards.

Contrary to the study of leopards in Thailand (Ngoprasert, Lynam & Gale, 2017) and Africa (Havmøller et al., 2019), our results indicate the frequent use of roads and disturbed habitats by leopards. Our results suggest that tigers and leopards in human-dominated areas frequently occur in degraded habitats relying partially on domestic prey species. Athreya et al. (2013) found a similar pattern of density estimates of leopard (4.8 ± 1.2) in human-dominated regions where leopards relied on domestic prey intake. The density estimates of leopard in this study are higher than the lowest estimates of leopard density (0.74 ± 0.18) recorded in India using the secr framework (Noor et al., 2020). Overall, the density estimates of tigers and leopards were lower compared to other studies in India. Karanth & Nichols (1998) estimated tiger densities to be (11.7 ± 1.93) in Kanha, (16.8 ± 2.96) in Kaziranga, (11.5 ± 1.70) in Nagarahole and (4.1 ± 1.31) in Pench Tiger Reserve. However, these estimates were calculated from the core zones that are relatively free from human interference.

Moreover, the estimates were calculated using traditional non-spatial CR methods that depend on extra buffer W around trapping grids. Recently, Sharma et al. (2010) compared the tiger densities using spatial likelihood methods in program density and using MMDM, 1/2MMDM as buffer strip, and based on home range radius calculated from telemetry, and found that densities were overestimated using 1/2MMDM. It is argued that in the absence of telemetry based home ranges, the spatial capture histories of camera traps should be used in likelihood-based density estimation methods (Borchers & Efford, 2008; Efford, Borchers & Byrom, 2008; Royle et al., 2008). The density estimation methods based on spatial likelihood approaches (SECR) do not depend on buffer widths around camera trap arrays. Thus the density estimates are least biased (Efford, 2004; Foster & Harmsen, 2012).

The observed density of tigers and leopards in the buffer zone may be regarded as moderate and not too low or high for a disturbed habitat. A literature survey indicated a huge disparity in the density estimates of leopards in India, varying from as low as 0.74 ± 0.18 (Noor et al., 2020) to the 28.9 ± 7.2 per 100 km^2^ reported in Mudumalai (Kalle et al., 2011). The densities of tigers in India have been found to correlate with the prey densities (Karanth & Nichols, 1998; Karanth et al., 2004a). The observed density estimates in the buffer zones are likely. Leopards have been reported to occur relatively at a higher density in human-dominated landscapes (Athreya et al., 2007, 2013) in the absence of tigers. Athreya et al. (2013) reported that leopards occurred at a density of 4.8 ± 1.2 individuals per 100 km^2^ in western India’s human-dominated landscapes. Kalle et al. (2011) reported a comparatively high density of leopards using SECR maximum likelihood (13.17 ± 3.15) and Bayesian models (13.01 ± 2.31) per 100 km^2^ in Mudumalai Tiger Reserve. Leopards consume a wide variety of prey species, and even in prey deficient habitats are reported to occur at moderate densities by consuming small to medium-sized prey species (Hayward et al., 2006; Athreya et al., 2013).

### Sampling adequacy

A maximum of 25 camera trap pairs were placed for eight consecutive days at any sampling occasion in 2 × 2 km grids before moving them to new locations. The presence of agricultural fields and human habitation within the buffer zone limited our inter-trap distance to 850 m. It is reported that in case of low tiger density (<2 per 100 km^2^), estimates of N can be achieved by increasing trap density. However, the estimates are likely to lack precision (Sharma et al., 2010). Silver et al. (2004) recommended the systematic approach of trap placement in medium to high tiger density areas with at least four trap nights km^2^ and greater than 50 camera traps per 100 km^2^. They also recommended a minimum sampling effort of over eight trap nights km^2^. While studying ocelots, Maffei & Noss (2008) suggested that camera trap grids should cover at least a minimum of three to four average home ranges. The placement of camera traps in 4 km^2^ grids spread over more than 500 km^2^ of the trapping area and thus would have provided a fair chance for all tigers and leopards to be photo-captured. The 95 detections of 33 individual tigers and 74 detections of 38 individual leopards over 2,211 trap nights within the study area also indicate the area’s adequate sampling.

## Conclusion

Spatially Explicit Capture–Recapture framework provides a robust approach to arriving at reliable density estimates of cryptic and wide-ranging carnivores. SECR framework can be used to assess the actual correlates of carnivore density by modeling the habitat covariates likely believed to influence the density estimates. Our study indicates that the variables described as a proxy of disturbance influence large carnivores’ density estimates in the human-dominated buffer regions around protected areas. The large carnivores in buffer regions may attain moderate densities by consuming the domestic prey species, which occur at high abundance around India’s protected areas. The human-dominated areas may act as essential refugee habitats for large and endangered carnivore species in the future. Thus it is crucial to maintain the wild ungulate prey species at high abundances in prey-deficient regions around protected areas.

## Supplemental Information

10.7717/peerj.10634/supp-1 Supplemental Information 1 A set of eight habitat covariates used to model the density of tiger and leopard. The variables were derived using the LULC map of the study area at 1 km spatial resolution.

10.7717/peerj.10634/supp-2 Supplemental Information 2 The arrangement of camera traps, capture-recapture locations, and the tracks of tiger and leopard detected in camera traps. Camera traps are represented by red crosses; the colored dots represent the unique individuals of tiger (on left panel) and leopard (on right panel). The lines connecting the dots represent the tracks.

10.7717/peerj.10634/supp-3 Supplemental Information 3 The Capture file of tiger used for the density estimation of tiger in SECR. First column represents the sessions; second column represents the unique ID of the individual tigers captured in camera traps; third colum represents the occasion on which anmals weres captured and fourth column represents the ID of the detectors (camera traps).

10.7717/peerj.10634/supp-4 Supplemental Information 4 The Capture file used for the density estimation of leopards in SECR. First column represents the sessions; second column represents the unique ID of the individual leopards captured in camera traps; third colum represents the occasion on which anmals weres captured and fourth column represents the ID of the detectors (camera traps).

10.7717/peerj.10634/supp-5 Supplemental Information 5 The trap layout file including eight habitat covariates used to model the densities of tiger and leopard. First column represents the camera traps; second and third columns reprsents the GPS cordinates of the camera traps in meters.

10.7717/peerj.10634/supp-6 Supplemental Information 6 Formal analysis of the spatial capture recaptures data of tigers and leopards. Where *n* = number of distinct individuals detected on each occasion *t*, *u* = number of individuals detected for the first time on each occasion *t*, *f* = number of individuals detected on exactly *t* occasions, and M (t+1) = cumulative number of detected individuals on each occasion *t*.

10.7717/peerj.10634/supp-7 Supplemental Information 7 Stabilization of density (rigid values) with respect to the buffer sizes. In tiger (A) and leopard (B) the half normal detection function reaches plateau fairly promptly compared to Hazard rate (HR) and Exponential (EX) detection function.

## Additional Information and Declarations

### Field Study Permissions

The following information was supplied relating to field study approvals (i.e., approving body and any reference numbers): The permission to collect the data at the field site was provided by the Madhya Pradesh, Forest Department, and the data collection was undertaken with the cooperation of Bandhavgarh Tiger Reserve administration.

### Data Availability

The following information was supplied regarding data availability: Raw data showing the capture histories of tiger and leopard are available in the Supplemental Files.
