# Supplementary material for: Density estimation of tiger and leopard using spatially explicit capture–recapture framework
Source: PeerJ. 2021 Feb 17;9:e10634. doi: 10.7717/peerj.10634 (PMC7896501; doi:10.7717/peerj.10634)
Supplement: Supplemental Information 1 — The variables were derived using the LULC map of the study area at 1 km spatial resolution. [file peerj-09-10634-s001.docx]

**Supplementary Table S2: A set of eight habitat covariates used to model the density of tiger and leopard**

| **Variable type** | **Variable** | **Abbreviation** |
| --- | --- | --- |
| Forest type | Sal dominated forest | sal |
|  | Moist deciduous forest | mdec |
|  | Dry deciduous forest | drydec |
|  | River bodies | river |
| Human influenced | Human settlements | set |
|  | Road density | rd1km |
|  | Degraded forest | degraded |
| Topography | Terrain ruggedness | rug |
