# Supplementary material for: Density estimation of tiger and leopard using spatially explicit capture–recapture framework
Source: PeerJ. 2021 Feb 17;9:e10634. doi: 10.7717/peerj.10634 (PMC7896501; doi:10.7717/peerj.10634)
Supplement: Supplemental Information 2 — Camera traps are represented by red crosses; the colored dots represent the unique individuals of tiger (on left panel) and leopard (on right panel). The lines connecting the dots represent the tracks. [file peerj-09-10634-s002.docx]

**Supplementary S2: The arrangement of camera traps, capture-recapture locations, and the tracks of tiger and leopard detected in camera traps.**

**
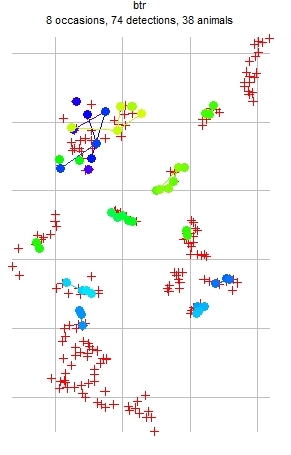

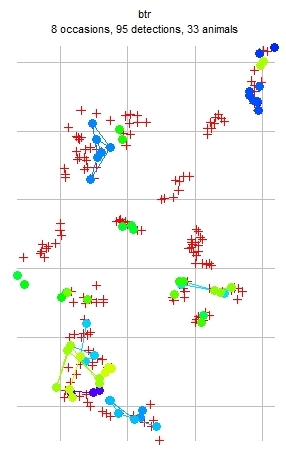
**

Camera traps are represented by red crosses; the colored dots represent the unique individuals of tiger (on left panel) and leopard (on right panel). The lines connecting the dots represent the tracks.
