# Supplementary material for: Density estimation of tiger and leopard using spatially explicit capture–recapture framework
Source: PeerJ. 2021 Feb 17;9:e10634. doi: 10.7717/peerj.10634 (PMC7896501; doi:10.7717/peerj.10634)
Supplement: Supplemental Information 6 — Where n = number of distinct individuals detected on each occasion t, u = number of individuals detected for the first time on each occasion t, f = number of individuals detected on exactly t occasions, and M (t+1) = cumulative number of detected individuals on each occasion t. [file peerj-09-10634-s006.docx]

**Supplementary S6: Formal analysis of the spatial capture recaptures data of tigers and leopards.**

**Summary of the capthist object constructed for analyzing the spatial capture recapture data of tigers using package ‘secr’**

| **Occasions** | **1** | **2** | **3** | **4** | **5** | **6** | **7** | **8** | **Total** |
| --- | --- | --- | --- | --- | --- | --- | --- | --- | --- |
| **n** | 9 | 10 | 9 | 10 | 12 | 6 | 9 | 7 | 72 |
| **u** | 9 | 9 | 3 | 6 | 2 | 0 | 4 | 0 | 33 |
| **f** | 13 | 9 | 8 | 0 | 2 | 0 | 1 | 0 | 33 |
| **M(t+1)** | 9 | 18 | 21 | 27 | 29 | 29 | 33 | 33 | 33 |
| **Detections** | 11 | 12 | 15 | 12 | 16 | 10 | 12 | 7 | 95 |
| **Detectors visited** | 11 | 10 | 14 | 11 | 14 | 9 | 12 | 6 | 87 |

**Summary of the capthist object constructed for analyzing the spatial capture recapture data of leopards using package ‘secr’**

| **Occasions** | **1** | **2** | **3** | **4** | **5** | **6** | **7** | **8** | **Total** |
| --- | --- | --- | --- | --- | --- | --- | --- | --- | --- |
| **n** | 12 | 7 | 8 | 9 | 6 | 10 | 7 | 8 | 67 |
| **u** | 12 | 7 | 3 | 5 | 3 | 3 | 3 | 2 | 38 |
| **f** | 17 | 15 | 5 | 0 | 1 | 0 | 0 | 0 | 38 |
| **M(t+1)** | 12 | 19 | 22 | 27 | 30 | 33 | 36 | 38 | 38 |
| **Detections** | 16 | 7 | 8 | 9 | 7 | 10 | 7 | 10 | 74 |
| **Detectors visited** | 15 | 7 | 6 | 9 | 6 | 10 | 7 | 10 | 70 |

Where n = number of distinct individuals detected on each occasion *t*, u = number of individuals detected for the first time on each occasion *t*, f = number of individuals detected on exactly *t* occasions, and M (t+1) = cumulative number of detected individuals on each occasion *t*

The cumulative number of detected individuals (M (t+1) in case of tigers reaches 33 individuals on 7^th^ occasion (Table S1) and does not change thereafter. However in case of leopards (Table S2) the cumulative number of detected individuals (M (t+1) does not stable even at 8^th^ sampling occasion. However, it is generally argued that camera traps fail to detect all the individuals even after prolonged sampling (Sharma et al. 2010).
