# Supplementary material for: Density estimation of tiger and leopard using spatially explicit capture–recapture framework
Source: PeerJ. 2021 Feb 17;9:e10634. doi: 10.7717/peerj.10634 (PMC7896501; doi:10.7717/peerj.10634)
Supplement: Supplemental Information 7 — In tiger (A) and leopard (B) the half normal detection function reaches plateau fairly promptly compared to Hazard rate (HR) and Exponential (EX) detection function. [file peerj-09-10634-s007.docx]

**Supplementary S7: Stabilization of density (rigid values) with respect to the buffer sizes. In tiger (panel A) and leopard (Panel B) the half normal detection function reaches plateau fairly promptly compared to Hazard rate (HR) and Exponential (EX) detection function**

**
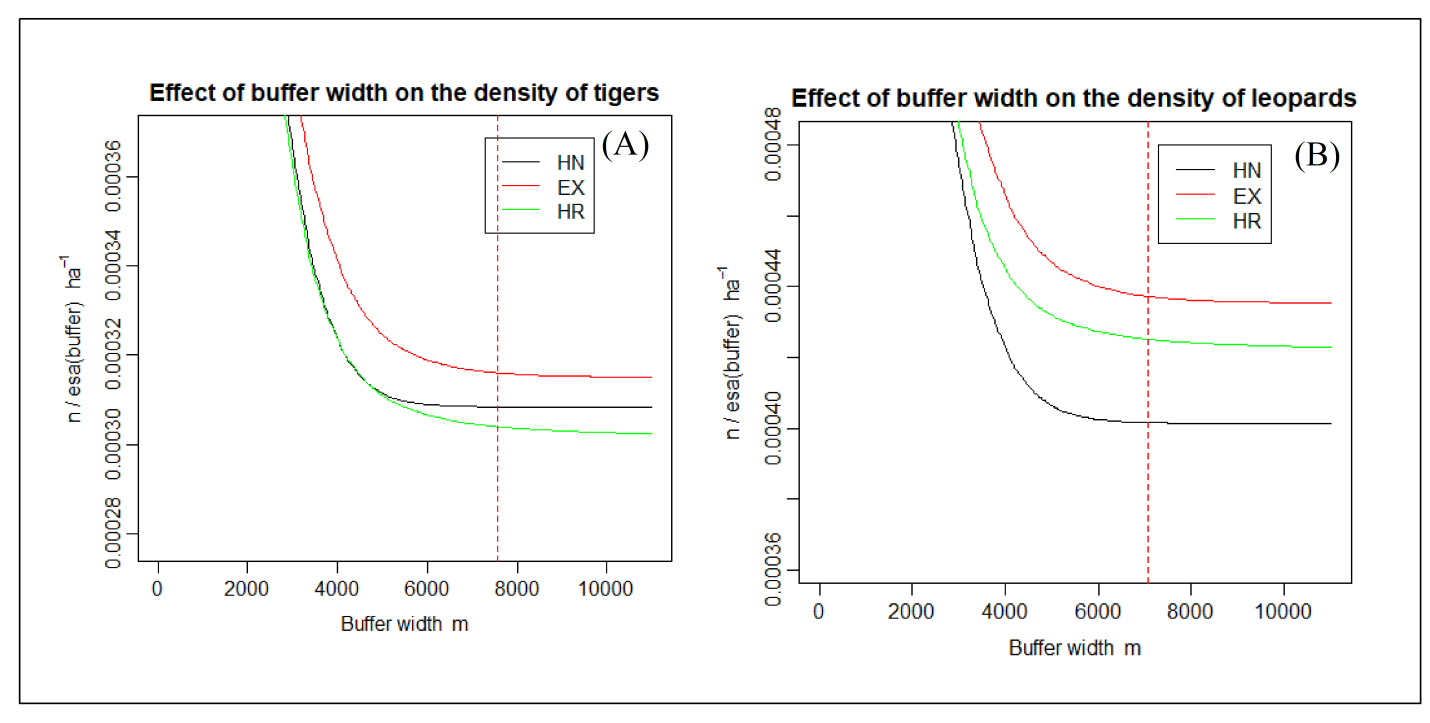
**
